# A qualitative exploration of deterrents to COVID-19 vaccination uptake among adults in post-war Tigray, Northern Ethiopia

**DOI:** 10.1038/s41598-025-28879-6

**Published:** 2025-12-12

**Authors:** Ferehiwot Hailemariam Tesfa, Znabu Hadush Kahsay, Tadele Tesfean, Adhena Ayaliew Werkneh, Brhane Ayele, Tsegay Hadgu, Hailay Gebretnsae, Moges Mekonnen, Ataklti Fisseha, Yaynshet Gebreyohannes, Joy Kenyi, Hnin Su Mon, Hayelom Kahsay, Ashenafi Asmelash, Gebrehaweria Gebrekurstos, Mussie Alemayehu, Araya Abrha Medhanyie

**Affiliations:** 1 School of Public Health, Mekelle University, College of Health Sciences, Mekelle, Tigray Ethiopia; 2 MARCH Research Center, College of Health Sciences, Mekelle, Tigray Ethiopia; 3 Tigray Health Research Institute, Mekelle, Tigray Ethiopia; 4 Tigray Health Bureau, Mekelle, Tigray Ethiopia; 5 UNICEF, Tigray Field Office, Mekelle, Tigray Ethiopia; 6 Mums for Mums, Mekelle, Tigray Ethiopia

**Keywords:** Diseases, Risk factors

## Abstract

Coronavirus disease 2019 (COVID-19) has had a profound impact on developing countries and continues to pose a serious global threat. Vaccination is essential for protecting public health, yet vaccine hesitancy remains a significant barrier to uptake. Evidence on the deterrents to COVID-19 vaccination in post-war contexts, particularly in Ethiopia, is limited. This study explored the factors hindering COVID-19 vaccine uptake in post-war Tigray, Northern Ethiopia. An exploratory qualitative study was conducted in Tigray from August 1 to 30, 2023. Six focus group discussions (FGDs) and seven in-depth interviews (IDIs) were held in host communities and internally displaced persons (IDP) centers, along with seven key informant interviews (KIIs) with public health experts. Participants were purposively selected, and data were collected using semi-structured guides refined through immediate debriefing. All sessions were audio-recorded, transcribed verbatim, and analyzed inductively using Atlas.ti (version 9.0). Five themes emerged as deterrents to COVID-19 vaccination uptake in post-war Tigray: perceived risk of COVID-19, reliance on cultural practices, post-war-related hindrances, infodemic-related barriers, and limited vaccine accessibility. The most frequently reported deterrents were low perceived risk, post-war challenges, fear of side effects, and accessibility issues, whereas reliance on cultural practices was mentioned less often. Low COVID-19 vaccine uptake in post-war Tigray is driven by individual perceptions, sociocultural beliefs, logistical barriers, and post-war challenges. Strengthening uptake requires engaging local religious leaders, implementing gender-sensitive campaigns addressing fertility concerns, and integrating vaccination with humanitarian food aid or health outreach programs.

**Supplementary Information:**

The online version contains supplementary material available at 10.1038/s41598-025-28879-6.

## Introduction

Coronavirus disease 2019 (COVID-19) has emerged as a global pandemic and remains a critical public health priority. Its rapid spread has placed substantial strain on health systems, caused millions of illnesses and deaths, and severely impacted economies worldwide^1^. According to World Health Organization (WHO) data, 777,026,543 confirmed COVID-19 cases and 7,078,481 deaths were reported between December 31, 2019, and December 8, 2024^2^.

Developing countries have borne a disproportionate burden from the pandemic and are likely to continue facing severe impacts unless a larger proportion of the global population is vaccinated^3,4^. Like many countries, Ethiopia has sought to control COVID-19 through various strategies, including mass vaccination with second and booster doses^5^. The country reported its first COVID-19 case on 13 March 2020. According to the WHO Coronavirus (COVID-19) Dashboard, by 10 September 2023, Ethiopia had recorded 500,872 confirmed cases and 7,574 deaths^1^.

Mass immunization is the primary strategy to control COVID-19 and achieve herd immunity^4,6^. Despite the well-documented benefits of vaccines, hesitancy remains a significant barrier to uptake^7^. Vaccine hesitancy arises from a complex interplay of contextual, individual, and vaccine-specific factors. The WHO SAGE Working Group on vaccine hesitancy framework highlights three key determinants—confidence, complacency, and convenience—that shape individual vaccination decisions^8^. Motivation to receive the COVID-19 vaccine is influenced by concerns about both short- and long-term side effects, as well as personal risk perception^9–12^. Limited knowledge of the risks of remaining unvaccinated and the benefits of vaccination further contributes to hesitancy^13–16^. Misconceptions and myths also amplify anti-vaccine sentiments in the population^7,11^.

Various social and economic factors can hinder vaccination efforts, particularly in war-affected areas where insecurity limits access to vulnerable populations^17–19^. Armed conflicts negatively affect the availability, quality, and uptake of vaccination services, often contributing to outbreaks of vaccine-preventable diseases (VPDs)^20,21^. In post-war and conflict-affected zones, poor compliance with COVID-19 mitigation strategies, including vaccination, further elevates community risk^22–24^.

Before the outbreak of armed conflict, Tigray had recorded over 6300 confirmed COVID-19 cases as of October 2020^25^. The conflict, which began in November 2020 during the ongoing pandemic, persisted for more than two years^26^. Although early pandemic containment efforts in Tigray had been relatively successful, with lower positivity and fatality rates, the war severely disrupted these gains. Health facilities were attacked, healthcare workers displaced, and service delivery interrupted, rendering COVID-19 prevention strategies nearly impossible to implement^24^.

The combined effects of the COVID-19 pandemic and armed conflict significantly worsened the public health crisis in Tigray^27^. Mitigation programs initiated at the onset of the outbreak were largely dismantled during the protracted conflict and siege^28^. Following a peace agreement in November 2022, a two-phase vaccination campaign reached approximately 1.3 million people, prioritizing internally displaced persons (IDPs) and other vulnerable populations^26^.

The post-war context in Tigray provides a unique setting to examine COVID-19 vaccine behavior. The armed conflict led to the collapse of health infrastructure, disruption of essential services, widespread displacement, and severe humanitarian crises. These structural challenges, combined with entrenched sociocultural beliefs, have created barriers to vaccination that are not captured in general vaccine hesitancy studies. To address these complexities, we employed a qualitative approach, enabling in-depth exploration of how perceptions, cultural beliefs, logistical barriers, and post-conflict conditions interact to shape vaccine uptake. By situating vaccine behavior within this fragile humanitarian context, the study provides context-specific insights that contribute to the literature and inform strategies to enhance COVID-19 vaccination coverage in post-war settings.

## Methods and participants

### Study setting and period

The study was conducted in the Tigray region of northern Ethiopia, which is bordered by Eritrea to the north, Sudan to the west, the Amhara region to the south, and the Afar region to the east. The region has a total population of 7,969,000^29^ and is administratively divided into seven zones (Mekelle, East, South, South East, North West, Western, and Central) and 93 districts (36 urban and 57 rural). The health system in Tigray comprises two specialized comprehensive hospitals, 14 general hospitals, 24 primary hospitals, 231 health centers, and 743 health posts.

**Supplementary Figure1:**
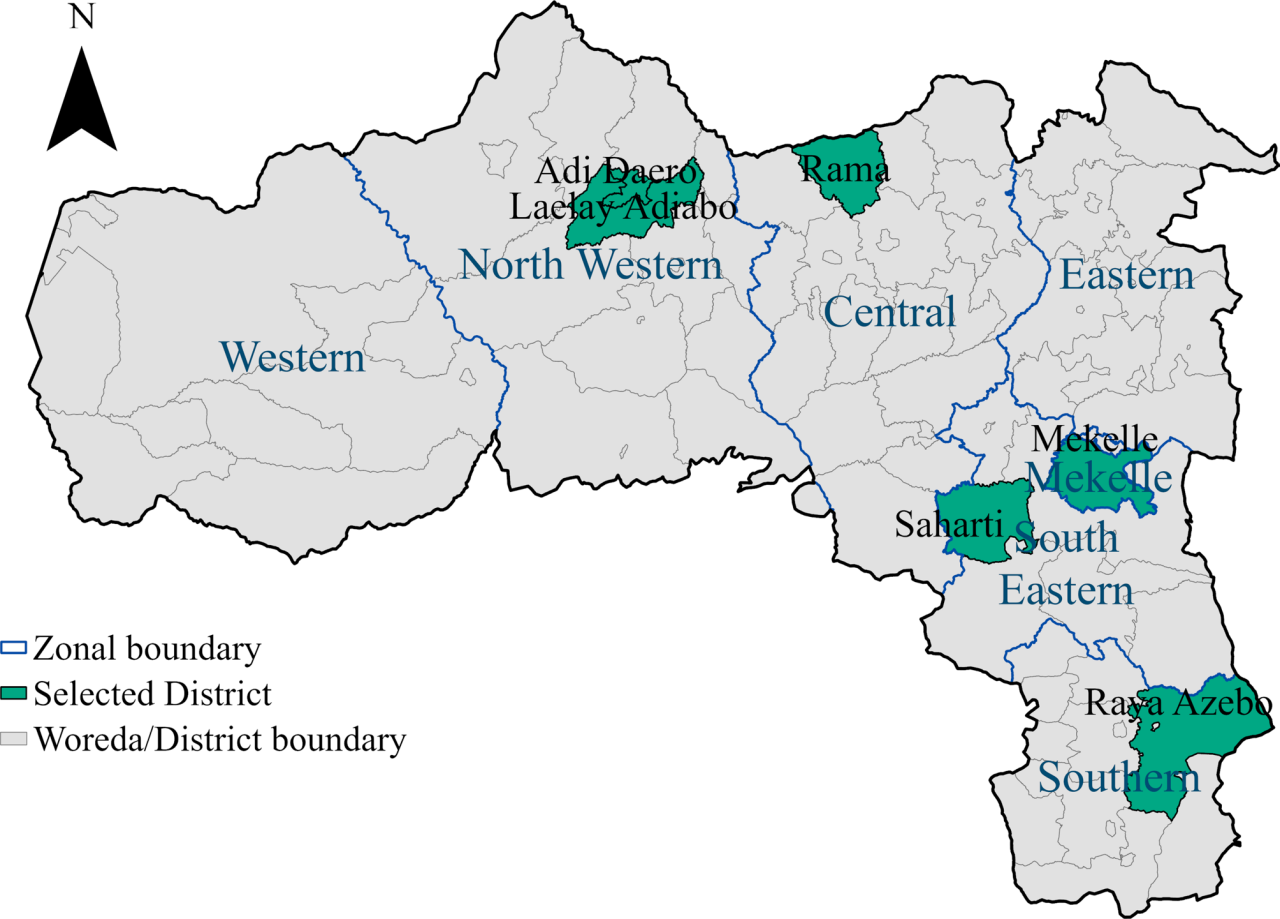
Map of the study area in Tigray region, Northern Ethiopia, showing seven purposively selected districts and one IDP center; five districts are individually highlighted, while two districts (Hawelti and Hadnet) and the IDP center in Mekelle zone are generally highlighted. The map was created by the authors using the “sf” package in R version 5.1 (https://www.R-project.org/) with shape files from The Humanitarian Data Exchange (https://data.humdata.org/dataset/cod-ab-eth).

The study was part of a larger research project on COVID-19 vaccination and maternal and child health, titled Integrated Health Survey on Utilization of Maternal and Child Health Services and COVID-19 Vaccination in Tigray Region, Northern Ethiopia, 2023^30^, conducted across 19 districts (woredas) from six zones of the region, excluding the western zone due to security concerns. For this qualitative component, seven districts (Hawelti, Adi Daero, Seharti, Raya Azebo, Rama Adi-Arbate, Laelay Adyabo, and Hadnet) and one IDP center were purposively selected from five zones (see supplementary Figure 1). Two districts (Hawelti and Hadnet) and the IDP center were selected from the Mekelle zone and are generally highlighted within this zone on the map. Data were collected between 1 and 30 August 2023.

### Study design

An exploratory qualitative study using focus group discussions (FGDs), in-depth interviews (IDIs), and key informant interviews (KIIs) was conducted to gain an in-depth understanding of factors deterring COVID-19 vaccine uptake among adults in post-war Tigray, Northern Ethiopia^31^. This design was selected due to the limited research on vaccine acceptance in conflict-affected settings and the lack of knowledge on behavioral, contextual, and structural factors influencing vaccination uptake in the region. The exploratory approach also enabled inductive identification of themes and patterns from participants’ experiences and perspectives.

### Source population

FGD and IDI participants included male and female adults residing in host communities and IDP settlements. KII participants comprised supervisors of health extension workers (the frontline workforce in the Ethiopian health system), health workers at health centers and primary hospitals involved in coordinating COVID-19 prevention activities, and experts from key partner organizations.

### Sampling and participants

The study included six FGDs: five with adults from host communities across five zones and one with participants from an IDP center, reflecting the relative population distribution in the study area. Each group comprised 8–10 participants and was conducted separately by gender to facilitate open dialogue. Participants were selected to represent a range of adult age groups, ensuring diverse perspectives. In addition, seven IDIs were conducted (four with male and three with female participants), along with six KIIs involving healthcare workers and other experts. Displacement status (IDP vs. host community), gender, and age were explicitly considered during participant selection to ensure balanced representation and capture potential variations in attitudes toward COVID-19 vaccination.

We employed the Maximum Variation Technique (MVT), a purposive sampling strategy designed to capture a wide range of perspectives^32^, by selecting participants who varied in sex, age, residence, and community type (host or IDP). MVT guided the selection of FGD participants across five zones, while maintaining relative homogeneity within each group in terms of gender and location to promote open discussion. KII participants were purposively selected for their depth of knowledge and expertise on COVID-19 vaccination, based on their professional roles and responsibilities.

### Data collection tools and procedure

Semi-structured guides for FGDs, IDIs, and KIIs were developed by a multidisciplinary team of experts in Health Promotion and Behavioral Sciences, Reproductive, Maternal, and Child Health (RMNCH), and Health Systems. The team, with prior experience in qualitative research, ensured that the guides were methodologically sound and aligned with the study objectives. Primary and probing questions were designed to elicit participants’ perspectives, clarify responses, and capture additional insights. The interview and discussion guide used for data collection are provided as Supplementary File 1.

The guides were refined through ongoing debriefings within the research team, allowing flexibility and continuous modification during data collection to integrate emerging insights. Interview questions were organized into two themes: (1) adults’ perspectives on deterrents to COVID-19 vaccination uptake, and (2) practical challenges encountered in receiving COVID-19 vaccinations in the post-war context. Data collection and analysis were conducted simultaneously, enabling the incorporation of new insights into revised versions of the guides.

All data collectors were male and female public health professionals trained in qualitative interviewing. They approached participants and selected suitable locations near participants’ homes or workplaces to ensure privacy, maintain confidentiality, and enable uninterrupted audio recording. Participants were consulted to determine convenient times for interviews to avoid conflicts with other tasks. For FGDs, community members were visited one day before the discussions. Potential participants were identified through community entry points, including health extension workers, community leaders, and village leaders. Based on the inclusion criteria, 8–10 individuals were selected and invited to participate the following day. All approached participants consented to take part, and none withdrew during interviews or discussions.

FGD participants were grouped homogeneously based on key characteristics (e.g., sex, residence, occupation, and community or religious roles) while allowing slight variations in other attributes to encourage diverse perspectives. To maintain confidentiality, participants were assigned ID codes (e.g., Code 1 to Code 10), avoiding the use of personal names during discussions. The number of FGDs, IDIs, and KIIs was guided by the principle of data saturation. Recruitment continued until no new codes or themes emerged, with saturation reached after the sixth FGD, seventh IDI, and sixth KII. Additional data collection beyond this point confirmed the consistency of identified themes.

To enhance reflexivity and minimize bias, the team held regular meetings during data collection to discuss emerging codes and assess the appearance of new information. Each FGD lasted approximately 90 min, while IDIs and KIIs lasted at least 45 min. No repeat interviews or discussions were conducted. All sessions were audio-recorded, and detailed field notes were maintained for each discussion and interview.

### Data analysis and management

All audio-recorded interviews and discussions were transcribed verbatim, with each transcription saved as a separate Microsoft Word file. Transcripts were then imported into Atlas.ti version 9.0 for systematic coding and thematic analysis.

The research team included investigators affiliated with local academic institutions and humanitarian agencies in the Tigray region. To address potential bias arising from these affiliations, investigators critically reflected on their positionality, prior knowledge, expectations, and experiences in the post-war context. Ongoing reflexive team discussions were conducted throughout data collection, transcription, coding, and analysis to identify and bracket potential biases. These measures ensured that findings were grounded in participants’ perspectives rather than preconceptions. An inductive qualitative approach was employed, and analysis was guided by thematic analysis principles.

Two members of the research team collaboratively developed a coding manual, which included a list of inductively generated codes, their definitions, and associated concepts. The manual was created through iterative reading and re-reading of transcripts and refined continuously throughout the coding process to ensure clarity and consistency. It was subsequently used to guide systematic coding of all transcripts imported into the qualitative data analysis software.

The data analysis team regularly reviewed the generated codes and reached consensus on renaming, merging, splitting, or deleting codes as needed. Similar codes were iteratively grouped into categories, which subsequently evolved into themes and sub-themes. For example, the participant statement, “Garlic is the vaccination for COVID-19. It is possible to prevent COVID-19 by putting garlic on one’s nose. In addition, we can also prevent it by steaming hot water,” was coded as ‘local remedies,’ grouped under the category ‘reliance on traditional remedies,’ and ultimately subsumed under the theme ‘cultural and spiritual practices.’ Similarly, the statement, “Many people seem to have forgotten about it. Furthermore, numerous individuals in our community believe the disease has disappeared; therefore, they do not intend to get vaccinated,” was coded as ‘perceived corona disappearance,’ clustered under the category ‘the time of COVID-19 has already passed,’ and incorporated into the theme ‘perceived COVID-19 risk.’ A second-line review was conducted to ensure that the emergent themes and sub-themes were well-grounded in participants’ statements. Due to logistical constraints in the post-conflict setting, transcripts and preliminary themes were not returned to participants for verification.

**Supplementary Figure2:**
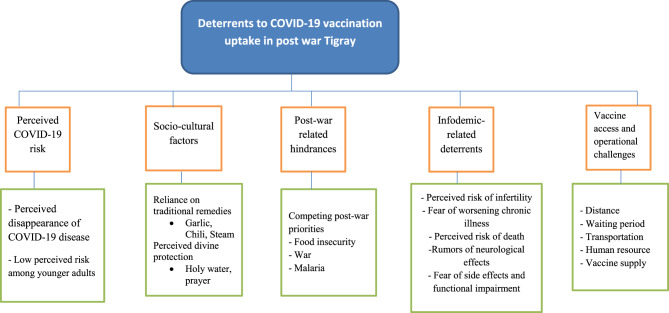
Themes and sub-themes of deterrents to COVID-19 vaccine uptake among adults in post-war Tigray, Northern Ethiopia.

Codes and quotations for each theme were extracted and reviewed to map salient concepts and establish a logical order for report writing. The final report included a summary of themes and sub-themes, accompanied by a detailed description of each. Illustrative quotes were selected to enrich the findings and ensure participants’ perspectives were conveyed in their own words. To guide interpretation within the study context, the WHO Behavioral and Social Drivers (BeSD) framework was applied, focusing on individuals’ thoughts and feelings, social processes, motivation, and practical factors influencing vaccine uptake^33^. To enhance transparency, a thematic map illustrating the main themes and sub-themes is provided in supplementary Figure 2. The study was reported following the COREQ (Consolidated Criteria for Reporting Qualitative Research) checklist.

## Results

### Participants’ socio-demographic characteristics

Adult participants were aged 22–68 years and were recruited from seven districts and one IDP center across five zones in the Tigray region. A total of six FGDs (five in host communities and one in an IDP center), seven IDIs, and six KIIs were conducted (Table 1).

**Table 1:** Sociodemographic characteristics of study participants.

Method	Number of participants	Gender	Age range	Educational status	Additional notes
FGDs	6 FGDs (n = 55)	4 male, 2 female groups	21–77 years	Mostly no formal education	Five FGDs were conducted in host communities and one in an IDP center.
IDIs	7	4 males, 3 females	22–68 years	No formal education to the secondary level	Six IDIs were conducted in host communities and one in an IDP center.
KIIs	6	3 males, 3 females	26–42 years	First degree and above	KIIs were conducted with both health workers and health experts.

### Description of themes

Five themes emerged as deterrents to COVID-19 vaccination uptake in post-war Tigray: (1) perceived COVID-19 risk, (2) reliance on spiritual and cultural practices, (3) post-war–related hindrances, (4) infodemic-related deterrents, and (5) vaccine accessibility and operational challenges (Table 2).

**Table 2:** Themes and sub-themes of deterrents to COVID-19 vaccine uptake among adults in post-war Tigray, Northern Ethiopia.

Themes	Subthemes
Theme 1: Perceived COVID-19 risk	“The time of COVID-19 has already passed.” “Younger adults face minimal risk of COVID-19 infection.”
Theme 2: Spiritual and cultural practices	Belief in traditional remedies Reliance on divine protection
Theme 3: Post-war-related hindrances	Competing post-war priorities
Theme 4: Infodemic-related deterrents and fear of side effects	Perceived risk of infertility and gendered concerns Fear of worsening chronic illness Perceived risk of death Rumors of neurological effects Fear of side effects and functional impairment
Theme 5: Vaccine accessibility and operational challenges	Distance and accessibility Campaign duration and scheduling Human resources and waiting time Vaccine supply

### Theme 1: Perceived COVID-19 risk

Many FGD and IDI participants perceived their risk of COVID-19 infection as low, which reduced motivation to receive the vaccine. Two main reasons were frequently cited: the belief that “the time of COVID-19 has already passed” and the perception that “younger adults face minimal risk of infection.”

"The time of COVID-19 has already passed": A recurring idea among participants was that the pandemic had largely subsided, diminishing urgency for preventive measures. Community concern had gradually faded, as illustrated by one young man:

*The severity of the disease appears to have decreased over time. Many people seem to have forgotten about it. Furthermore, numerous individuals in our community believe the disease has disappeared; therefore, they do not intend to get vaccinated* (22 years old, male, Hawelti district).

However, a minority of participants emphasized that this lowered risk perception did not necessarily reflect the actual situation. They noted that the ongoing post-war crisis had diverted attention from COVID-19 prevention. One farmer explained:

*In relation to the post-war situation, we started to overlook maintaining social distance. We neglected prevention mechanisms. Before this situation, we were washing our hands properly and eating alone to maintain social distance. Thus, we cannot say that we are free of the infection* (55 years old, male, Rama Adi-Arbaete district).

“Younger adults face minimal risk of COVID-19 infection”: Participants consistently noted that younger adults in their communities perceived themselves to be at low risk of contracting COVID-19 or experiencing severe illness. This sense of invulnerability was frequently cited as a reason for reduced vaccine uptake. As one young focus group participant remarked:

*Only a few young adults get vaccinated in our locality. This negligence among younger adults contributes to the overall low vaccination rates in this demographic* (21-year-old, female, Hawelti district).

In addition to age-related perceptions, some participants highlighted beliefs linked to identity that further reduced motivation for vaccination. Such narratives fostered a sense of invulnerability, reinforcing the tendency to underestimate the potential impact of COVID-19. A male focus group participant shared:

*Many people believe, 'Corona cannot harm or kill Habesha, but malaria and hunger are real enemies.' This belief underscores a dangerous underestimation of COVID-19’s potential impact, particularly among younger individuals who may feel invulnerable* (59-year-old, male, Rama Adi-Arbaete District).

### Theme 2: Spiritual and cultural practices

Most FGD participants, along with several IDI participants, reported relying on traditional and spiritual practices as primary means of preventing or managing COVID-19. These culturally grounded practices reduced the perceived need for vaccination, as individuals felt adequately protected through their own methods. The two most frequently cited approaches were belief in traditional remedies and reliance on divine protection.

“Belief in traditional remedies”: Participants explained that the use of natural ingredients, such as garlic, chili, and steam therapy, was widely accepted as a protective measure. This reliance on familiar remedies contributed to reduced motivation to seek vaccination. For example, a female focus group participant noted,

*Garlic is the vaccination for COVID-19. It is possible to prevent COVID-19 by putting garlic on one’s nose. In addition, we can also prevent it by steaming hot water* (35-year-old, female, Laelay Adyabo district).

Similarly, another participant added:

*As a prevention mechanism, just eat chopped garlic and chili together* (40-year-old, female, Hawelti District).

Reliance on divine protection: Several FGDs and a few IDIs revealed that community members frequently relied on spiritual practices, such as divine protection, as a primary means of preventing COVID-19. This belief often outweighed trust in medical interventions, including vaccination, and contributed to a reduced perceived necessity for vaccination. Participants noted that some community members preferred faith-based protection, including holy water, over seeking vaccines or medical care.

A female FGD participant explained:

*Those who are aware of the problem want to get vaccinated. However, for a considerable number of the population, divine protection is still the only thing they prefer. This is because we are not fully aware of the issue. Even if we get sick, rather than going to a health facility, we rely on holy water."* (21-year-old, female, Hawelti District)

A male participant added,

*Adults believe that only God can decide on their lives regardless of vaccination status against the coronavirus* (32-year-old, male, Seharti District).

### Theme 3: Post-war-related hindrances

Competing post-war priorities: Participants across FGDs, IDIs, and KIIs consistently reported that immediate post-war challenges, particularly food insecurity and ongoing insecurity, overshadowed concerns about COVID-19 and reduced motivation to vaccinate. Daily survival needs were prioritized over preventive health behaviors, even when participants were aware of the risks associated with COVID-19. This focus appeared to diminish perceived urgency regarding the pandemic and vaccine uptake. Many participants emphasized that survival needs, including access to food and protection from ongoing insecurity, were prioritized over pandemic-related risks.

As one health extension supervisor explained:

*For the people in our community, what is taking the lives of the youth is hunger, not COVID-19. What we urgently need is an effort to save our lives from hunger (*26-year-old, male).

FGD participants emphasized the perceived absence of visible consequences from COVID-19 infection. Several participants also noted concern over other concurrent health threats, including ongoing outbreaks and post-war challenges. One discussant summarized this perspective:

*In 2020, when COVID-19 was declared a global epidemic, everyone was trying to avoid gatherings. However, now, when you mention COVID-19, people respond with, 'Which COVID-19 are you talking about?' Currently, the concerns are hunger, insecurity, and malaria. There is no coronavirus at this time. What exists is the corona of hunger, the corona of post-war, and the corona of malaria! (*59-year-old, male, Mekelle IDP site*).*

### Theme 4: Infodemic-related deterrents and fear of side effects

Participants reported widespread misperceptions and fears that contributed to COVID-19 vaccine hesitancy and resistance. These concerns were often fueled by misinformation and community rumors. Reported fears included infertility, miscarriage, worsening of chronic illnesses, death, neurological problems, and other side effects.

Perceived risk of infertility and gendered concerns: Misperceptions and rumors linking COVID-19 vaccination to infertility critically shaped vaccination behavior in Tigray. Both men and women expressed concerns about fertility, but gender differences were particularly salient. Women highlighted pregnancy-related risks, delaying vaccination until after delivery. For instance, one woman stated:

*After my husband got vaccinated, he suggested that I also go for it, but I felt fearful and refused because I was pregnant. Later, I got vaccinated after giving birth* (26-year-old female, Seharti District).

Men also reported fears of infertility, although these concerns often reflected broader social narratives rather than personal reproductive risks. One farmer remarked:

*Some of us used to say, 'Who knows if it made us infertile permanently?' There are such concerns in the community* (62-year-old male, Seharti District).

Another participant shared a personal experience:

*It (the vaccine) made me sick for three days. After that, I was deeply concerned about the risk of being infertile forever* (55-year-old, male, Raya Azebo District).

A few others cited circulating rumors linking vaccination to the gender of the child:

*Nowadays, some people say, 'Those who get vaccinated for COVID-19 will only give birth to females.' That is what people have been saying* (35-year-old male, Raya Azebo District).

Fear of worsening chronic illness: Participants with chronic conditions, including diabetes, hypertension, and gastritis, expressed concern that vaccination could exacerbate their illnesses, despite understanding the vaccine’s benefits. One participant noted:

*I asked the healthcare providers if it would negatively affect me because I have gastritis. They assured me it wouldn’t, but I was worried at first* (55-year-old, female, Hawelti District).

Another focus group participant reflected broader community perceptions:

*We actually believe in the importance of the vaccine. However, there are concerns that it could seriously affect chronic patients and even cause death. This worry stems from instances where complications have occurred among those with conditions like high blood pressure and diabetes. Otherwise, we do believe in the vaccine* (62-year-old, male, Mekelle IDP site).

Perceived risk of death: Although no vaccine-related deaths were reported locally, rumors from neighboring areas fueled fear of fatal outcomes. One focus group discussant explained:

*We have been hearing from other areas about deaths related to the COVID-19 vaccine. However, it hasn’t happened in our Tabiya (the smallest administrative unit), yet some people still fear death associated with the vaccine* (27-year-old, female, Hawelti district).

Rumors of neurological effects: Several participants reported fears of severe neurological consequences, including epilepsy. A key informant observed:

*I have been hearing discouraging ideas like, 'If you get vaccinated, you will get epilepsy* (26-year-old, female, Seharti district).

Fear of side effects and functional impairment: Concerns about general side effects were prominent, particularly among older adults and those performing physically demanding work. Participants noted that post-war nutritional vulnerability and occupational demands amplified perceived risks, contributing to vaccine hesitancy. A male participant noted:

*It is said that the vaccine causes excessive sleepiness in the elderly. Since the post-war crisis began in 2020, many adults, especially elders, have weakened immune systems. Because of these effects, many refuse to get vaccinated (33-year-old male, Mekelle IDP site).*

Participants also expressed concern that side effects could interfere with daily functioning or work. A focus group discussant remarked:

*I have observed that for some people, the vaccine can be too powerful, and they struggle to handle the side effects. Some may need to stay in bed for a day or two* (28-year-old male, Seharti district).

A female millhouse worker highlighted occupational concerns:

*They told us that there is a vaccination program. At the beginning, we refused it, assuming it could interfere with my work type because I work close to a mill motor* (40-year-old female, Seharti district).

### Theme 5: Vaccine accessibility and operational challenges

Participants in FGDs and IDIs highlighted several structural and operational barriers that limited adults’ access to COVID-19 vaccination services. These included long travel distances, short campaign durations, limited human resources, prolonged waiting times, and vaccine shortages, all of which contributed to reduced uptake.

Distance and accessibility: Many participants reported that adults had to travel long distances to reach vaccination posts, particularly in rural areas with limited transportation, which discouraged vaccine uptake. One participant suggested:

*I would suggest that healthcare providers establish vaccination posts in the villages. This way, adults could get vaccinated more conveniently, without the hassle of walking long distances. It would be better if the vaccination posts were located at the health facilities within the respective tabiyas (the smallest administrative unit)* (22-year-old male, Hawelti district).

Campaign duration and scheduling: Short lead times for community mobilization and logistical preparation posed significant barriers. Vaccination campaigns were often dependent on external funding, which delayed planning and implementation. Compressed timelines left many adults unvaccinated for extended periods. One farmer explained:

*Additional days should be allocated for the campaign. For instance, if there are three tabiyas, an independent day should be designated for each one to ensure that no one is missed. Many adults do not get vaccinated because the number of days is too short* (49-year-old male, Laelay Adyabo district).

Human resources and waiting times: Limited healthcare personnel led to staff being overextended, resulting in long wait times and some adults leaving without being vaccinated. This issue was particularly pronounced when only a single day was allocated per village.

One participant described:

*The vaccination period allotted for each village was only one day. A single health provider can’t vaccinate so many people in that time. As a result, some individuals who came for vaccination had to leave without receiving it* (29-year-old female, Seharti district).

Extended waiting periods also created challenges for adults with caregiving responsibilities. Participants noted frustration and attrition due to long queues:

*The problem is sitting and waiting for the vaccination at the post. Since we need to be vaccinated, we sit and wait with all our worries about the kids at home. What else can we do?* (28-year-old female, Hawelti district).

Another focus group discussant added:

*People who come for vaccination may not receive it on time. Adults have many routine activities, and when they become impatient, they often leave the vaccination post without getting vaccinated. Adults should be able to attend to their daily tasks* (62-year-old male, Seharti district).

Vaccine supply: Participants reported that vaccine unavailability during campaign days further hindered access. Poor coordination of supply and transportation logistics left many willing adults unvaccinated. One participant noted:

*Even at the height of COVID-19 concerns, there were times when we were informed that no vaccines were available. I strongly suggest that the vaccine should be made available in adequate amounts* (28-year-old male, Seharti district).

## Discussion

This study explored deterrents to COVID-19 vaccine uptake among adults in post-war Tigray, northern Ethiopia. Overall, motivation to vaccinate was low and influenced by multiple, interrelated factors, including perceived COVID-19 risk, cultural and spiritual beliefs, competing post-war priorities, widespread misperceptions and rumors, fear of vaccine side effects, and limited access to vaccination services.

Adults perceived COVID-19 as a diminished threat, which contributed to reduced motivation to vaccinate. Interpreted through the WHO BeSD framework, this reflects complacency, where perceived personal risk is minimal. Low perceived threat can suppress proactive health behaviors. This finding aligns with studies from Ethiopia and other settings reporting low uptake among younger adults^11,16,34–37^, whereas some African communities have documented comparatively higher uptake, highlighting contextual differences in risk perception^38,39^. The prevailing belief that the virus poses minimal personal risk also echoes global evidence linking vaccine hesitancy to limited community health literacy^10,13^. Vaccination campaigns should therefore explicitly communicate ongoing risks, leveraging locally trusted voices to counter perceptions that the pandemic has “ended.”

In Tigray, survival needs—particularly hunger—took precedence over preventive measures such as vaccination. Persistent food insecurity following the war led communities to deprioritize vaccination in favor of meeting daily subsistence needs^40^. Although the risk of concurrent outbreaks of COVID-19 and malaria was acknowledged^24^, response efforts were undermined by the collapse of the public health system during the war and siege^41^. Within the WHO Behavioural and Social Drivers (BeSD) framework, these findings highlight that vaccine uptake is influenced not only by risk perception but also by structural barriers.

Comparable dynamics have been reported in other conflict-affected contexts. In Yemen, economic hardship, long travel distances, and livelihood pressures constrained access to vaccination^19^. In Syria, low perceived risk, fears of side effects, and disinformation shaped negative attitudes toward vaccines^20^. In the Democratic Republic of Congo, mistrust and reliance on misinformation undermined vaccine demand^42^. These cross-contextual parallels illustrate how both cognitive and structural domains of the BeSD framework converge to limit vaccine uptake in fragile settings. However, the Tigray case underscores the compounded effects of siege and extreme food insecurity, which uniquely intensified the deprioritization of COVID-19 vaccination. Integrating vaccination campaigns with ongoing humanitarian relief efforts may improve vaccine uptake in post-conflict settings.

Cultural and spiritual beliefs played a central role in shaping community attitudes toward COVID-19 vaccination in Tigray. Our findings highlighted reliance on garlic, chili, and steam inhalation as primary preventive measures. Similarly, reliance on herbal and traditional remedies has been documented in other low-resource African and Asian contexts^43–45^, underscoring how entrenched cultural practices influence health-seeking behavior during pandemics.

Perceived divine protection also emerged as a driver of vaccine hesitancy. Reliance on holy water and prayer persisted even after vaccines became available, reflecting stronger trust in spiritual practices than in biomedical solutions. These preferences may be reinforced in post-conflict settings, where institutional trust is eroded due to collapsed health services. Comparable evidence from Pakistan and Ethiopia demonstrates that religious beliefs can negatively affect vaccine acceptance^6,46^. These findings reflect a confidence gap in biomedical interventions and correspond to the confidence and social influence domains of the WHO BeSD framework. Similar patterns reported in Tanzania^47^ highlight that engaging religious leaders to disseminate accurate information can help bridge the gap between biomedical solutions and spiritual worldviews.

Perceived risk of adverse health effects was another significant deterrent, particularly among older adults. Concerns about fever, fatigue, and potential long-term health consequences—especially regarding fertility—were prominent. Globally, vaccine safety concerns have consistently emerged as barriers to uptake^10,37,48^. These concerns were often amplified by perceptions of the vaccine’s novelty and uncertainty regarding long-term effects, a pattern documented in other studies^16,49^. Evidence also suggests that trusted individuals within social networks who have been vaccinated and share positive experiences may help alleviate such concerns and promote vaccine acceptance^14,15,36,38^.

Our findings demonstrate that reproductive health myths disproportionately affect women, consistent with global evidence showing that pregnancy- and fertility-related fears reduce vaccine uptake among females^7,48,50^. Systematic reviews and studies from other contexts, such as Morocco, confirm that perceived maternal and fetal risks are key deterrents to vaccination^10,16,36,51^. These misconceptions span both the confidence and social processes domains of the BeSD framework, highlighting how gendered social norms and trust in health information intersect to influence behavior. Addressing vaccine hesitancy in post-war contexts, therefore, requires gender-sensitive communication strategies, including targeted messaging to dispel myths about fertility and pregnancy risks and community engagement that considers both male and female perspectives.

Limited accessibility to vaccination services emerged as a major deterrent. Long travel distances, extended waiting times, and short-duration vaccination campaigns posed practical challenges to receiving vaccines, reflecting constraints within the practical issues domain of the BeSD framework. Structural barriers were further compounded by reliance on external aid for vaccine supply and the collapse of the healthcare system during the war, which severely restricted access to essential health services^7,24^.

Comparable logistical challenges have been documented in other conflict-affected settings. In post-war Darfur, shortages of healthcare professionals hindered vaccine delivery^52^, and inadequate vaccine availability has limited the speed and coverage of campaigns in multiple low-resource contexts^53^. Studies also emphasize the importance of establishing accessible vaccination sites and extending campaign duration to improve vaccine uptake^6,36,37,48,54^. These findings underscore the need for context-specific interventions to address structural barriers, such as decentralized vaccination points and mobile clinics, to reach populations in hard-to-access areas in post-conflict settings.

### Strengths and limitations

This exploratory qualitative study provided in-depth insights into the deterrents to COVID-19 vaccination uptake in a post-war setting. The use of multiple data sources—FGDs, IDIs, and KIIs—enhanced the credibility of the findings through triangulation. Additionally, applying a maximum variation sampling technique allowed us to capture diverse perspectives across different community settings and demographic groups.

However, the study has some limitations. The COVID-19 vaccination campaign and data collection occurred soon after the cessation of hostilities; therefore, participants’ responses may have been influenced by the immediate post-war context. Their perceptions might evolve as the humanitarian situation stabilizes and access to information improves.

## Conclusions and recommendations

This study explored the multifaceted deterrents to COVID-19 vaccine uptake among adults in post-war Tigray, Northern Ethiopia, and used triangulated evidence from FGDs, IDIs, and KIIs. The converging thematic findings revealed that low vaccine uptake was driven by a complex interplay of individual perceptions, sociocultural beliefs, logistical barriers, and post-conflict conditions. Perceived low susceptibility to infection—particularly among younger adults—and the belief that the “time of COVID-19 has passed” substantially reduced motivation for vaccination. Misconceptions about severe side effects and reliance on religious alternatives further discouraged uptake. Structural challenges, including limited human resources, poor accessibility to vaccination sites, and insufficient preparation time for campaigns, also hindered coverage. The post-war humanitarian crisis, marked by food insecurity and competing priorities, further deprioritized COVID-19 prevention within communities.

To address these barriers in the post-war context, vaccination strategies should leverage existing community structures and humanitarian responses to ensure feasibility. Engaging local religious leaders can disseminate accurate information and bridge gaps between biomedical recommendations and spiritual beliefs. Gender-sensitive campaigns can address fertility-related concerns among women, while integrating vaccination with essential services—such as food aid or health outreach—can improve access where infrastructure is limited. Finally, future mixed-methods studies that incorporate quantitative assessments could further inform evidence-based, contextually appropriate interventions in post-conflict areas.

## Supplementary Information

Supplementary Information.

## Data Availability

Data are fully available from the corresponding author on reasonable request.

## References

[1] Cucinotta, D. & Vanelli, M. WHO declares COVID-19 a pandemic. *Acta Biomed.* **91**, 157–160 (2020). 32191675 10.23750/abm.v91i1.9397 PMC7569573

[2] World Health Organization. WHO Coronavirus (COVID-19) Dashboard. https://covid19.who.int (accessed 15 Sep 2024).

[3] Levin, A. T. et al. Assessing the burden of COVID-19 in developing countries: systematic review, meta-analysis and public policy implications. *BMJ Glob. Health* **7**, e008477 (2022). 35618305 10.1136/bmjgh-2022-008477 PMC9136695

[4] Yarlagadda, H. et al. COVID-19 vaccine challenges in developing and developed countries. *Cureus* **14**, e24215 (2022). 35547442 10.7759/cureus.23951 PMC9085716

[5] Gebreeyesus, M. Ethiopia’s response to the COVID-19 pandemic: measures, impacts and lessons. https://unctad.org/system/files/official-document/BRI-Project_RP25_en.pdf (2022).

[6] Muhammad, S. Z., Shaikh, N., Asad, D. & Fatima, N. Challenges to mass immunization against COVID-19 in Pakistan: a lower-middle-income vaccine-hesitant country. *Unpublished manuscript*.

[7] Hammad, A. M., Al-Qerem, W., Zaid, A. A., Khdair, S. I. & Hall, F. S. Misconceptions related to COVID-19 vaccines among the Jordanian population: myth and public health. *Disaster Med. Public Health Prep.* **17**, e207 (2023). 10.1017/dmp.2022.143 PMC9300972 35673791

[8] WHO Strategic Advisory Group of Experts (SAGE) on Immunization. In *Report of the SAGE Working Group on Vaccine Hesitancy* (World Health Organization, 2014).

[9] Mohammed, R. et al. COVID-19 vaccine hesitancy among Ethiopian healthcare workers. *PLoS ONE* **16**, e0261125 (2021). 34919597 10.1371/journal.pone.0261125 PMC8682893

[10] Nehal, K. R., Steendam, L. M., Campos Ponce, M., van der Hoeven, M. & Smit, G. S. Worldwide vaccination willingness for COVID-19: a systematic review and meta-analysis. *Vaccines* **9**, 1071 (2021). 34696179 10.3390/vaccines9101071 PMC8540052

[11] Kalayou, M. H. & Awol, S. M. Myth and misinformation on COVID-19 vaccine: the possible impact on vaccination refusal among people of northeast Ethiopia: a community-based research. *Risk Manag. Healthc. Policy* **15**, 1859–1868 (2022). 36213385 10.2147/RMHP.S366730 PMC9534150

[12] Valencia, O. A., Gonzalez, Y. B., Daza, J. S., & Villaquiran, A. F. Associated factors in the intention of vaccination against COVID-19, in Popayán, Cauca, Colombia. *Vacunas (Engl. Ed.)* **24**, 174–181 (2023). 10.1016/j.vacun.2023.01.003 PMC9842627 36685050

[13] Sammut, F., Suda, D., Caruana, M. A. & Bogolyubova, O. COVID-19 vaccination attitudes across the European continent. *Heliyon* **9**, e18610 (2023). 37588607 10.1016/j.heliyon.2023.e18903 PMC10425897

[14] Bui, H. N. et al. Utilizing the theory of planned behavior to predict COVID-19 vaccination intention: a structural equational modeling approach. *Heliyon* **9**, e16481 (2023). 37366521 10.1016/j.heliyon.2023.e17418 PMC10275777

[15] Toledo-López, A., Leyva-Hernández, S. N., Jiménez-Castañeda, J. C. & Avendaño-Rito, M. C. Determinants for COVID-19 vaccination intention in Mexico. *Heliyon* **9**, e18601 (2023). 37520986 10.1016/j.heliyon.2023.e18079 PMC10382286

[16] Wagner, A. et al. Let’s talk about COVID-19 vaccination: relevance of conversations about COVID-19 vaccination and information sources on vaccination intention in Switzerland. *Vaccine* **41**, 5313–5321 (2023). 37455160 10.1016/j.vaccine.2023.07.004

[17] DeLand, K. Vaccine equity in conflict-affected areas: the challenges of development, production, procurement, and distribution. *Unpublished report*.

[18] UN OCHA & UNCT Ukraine. Ukraine: 2018 Humanitarian Response Plan (January–December 2018). https://reliefweb.int/report/ukraine/ukraine-2018-humanitarian-response-plan-january-december-2018-enuk (2017).

[19] ACAPS. *COVID-19: Current situation and reasons for vaccine hesitancy.* ACAPS Analysis Hub, Thematic Report, 10 January 2022. https://www.acaps.org/fileadmin/Data_Product/Main_media/20220110_acaps_yemen_analysis_hub_thematic_report_covid-19_and_vaccine_hesitancy_0.pdf.

[20] Al-Abdulla, O., Alaref, M., Kallström, A. & Kauhanen, J. Individual and social determinants of COVID-19 vaccine hesitancy and uptake in Northwest Syria. *BMC Health Serv. Res.* **24**(1), 265. 10.1186/s12913-024-10756-z (2024). 38429739 10.1186/s12913-024-10756-z PMC10908183

[21] Ciccacci, F. et al. Between war and pestilence: the impact of armed conflicts on vaccination efforts: a review of literature. *Front. Public Health* **13**, 1604288 (2025). 40666147 10.3389/fpubh.2025.1604288 PMC12259657

[22] Fakhim, H. et al. Asymptomatic carriers of coronavirus disease 2019 among healthcare workers in Isfahan. *Iran. Future Virol.* **16**, 93–98 (2021).

[23] Abraha, H. E. et al. Impact of a double catastrophe, war and COVID-19, on health service utilization of a tertiary care hospital in Tigray: an interrupted time-series study. *Confl. Health* **17**, 5 (2023). 37580780 10.1186/s13031-023-00537-6 PMC10426210

[24] Gesesew, H. et al. The impact of war on the health system of the Tigray region in Ethiopia: an assessment. *BMJ Glob. Health* **6**, e007328 (2021). 34815244 10.1136/bmjgh-2021-007328 PMC8611430

[25] OCHA. Ethiopia: The pre-crisis situation in Tigray – Secondary Data Review, 22 February 2021. https://reliefweb.int/report/ethiopia/ethiopia-pre-crisis-situation-tigray-secondary-data-review-22-february-2021 (2021).

[26] World Health Organization. Reaching the unreached with COVID-19 lifesaving vaccination. https://www.afro.who.int/countries/ethiopia/news/reaching-unreached-covid-19-lifesaving-vaccination (accessed 3 Aug 2024).

[27] Gebru, K. T. et al. COVID-19 pandemic during the war in Tigray, Northern Ethiopia: a sequential mixed-methods approach. *Front. Public Health* **13**, 1553452 (2025). 40308932 10.3389/fpubh.2025.1553452 PMC12040842

[28] Vanden Bempt, T., Annys, S., Negash, E., Ghekiere, R. & Nyssen, J. Tigray: one year of conflict—casualties of the armed conflict, 2020–2021. Ghent University & Every Casualty Counts. https://www.everycasualty.org/reports/tigray-one-year-of-conflict (2021).

[29] Joshua Project. Tigray in Ethiopia. https://joshuaproject.net/people_groups/15481/et (accessed 2024).

[30] Alemayehu, M. et al. Does integration of health survey bring cost effectiveness into evidence generation in post-conflict settings of Tigray, Ethiopia?. *East Afr. J. Health Sci.* **6**, 798–808 (2024).

[31] Rendle, K. A., Abramson, C. M., Garrett, S. B., Halley, M. C. & Dohan, D. Beyond exploratory: a tailored framework for designing and assessing qualitative health research. *BMJ Open* **9**, e030123 (2019). 31462482 10.1136/bmjopen-2019-030123 PMC6720470

[32] Patton, M. Q. *Qualitative Evaluation and Research Methods* (SAGE Publications, 1990).

[33] World Health Organization. *Behavioral and Social Drivers (BeSD) of Vaccination: Tools and Guidance for Data Collection, Analysis, and Use* (WHO, 2022).

[34] Johannes, S. et al. COVID-19 vaccine hesitancy among adults in Hawassa City Administration, Sidama Region, Ethiopia: a community-based study. *Front. Public Health* **11**, 1122418 (2023). 36935692 10.3389/fpubh.2023.1122418 PMC10017993

[35] Viswanath, K. et al. Individual and social determinants of COVID-19 vaccine uptake. *BMC Public Health* **21**, 818 (2021). 33910558 10.1186/s12889-021-10862-1 PMC8081000

[36] Limbu, Y. B. & Gautam, R. K. The determinants of COVID-19 vaccination intention: a meta-review. *Front. Public Health* **11**, 1162861 (2023). 37377544 10.3389/fpubh.2023.1162861 PMC10291626

[37] Rios-Zertuche, D. *et al.* Factors associated with COVID-19 vaccination in Belize. *Vaccine: X* **15**, 100380 (2023). 10.1016/j.jvacx.2023.100380 PMC10483062 37693845

[38] Van Espen, M., Dewachter, S. & Holvoet, N. COVID-19 vaccination willingness in peri-urban Tanzanian communities: towards contextualising and moving beyond the individual perspective. *SSM Popul. Health* **22**, 101381 (2023). 36936725 10.1016/j.ssmph.2023.101381 PMC10014502

[39] Ojo, T. O. et al. Determinants of COVID-19 vaccine uptake among Nigerians: evidence from a cross-sectional national survey. *Arch. Public Health* **81**, 95 (2023). 37237389 10.1186/s13690-023-01107-1 PMC10214318

[40] Weldegiargis, A. W. et al. Armed conflict and household food insecurity: evidence from war-torn Tigray, Ethiopia. *Confl. Health* **17**, 22 (2023). 37147686 10.1186/s13031-023-00520-1 PMC10163686

[41] Abera, B. T. et al. Prevalence of COVID-19 and associated factors among healthcare workers in the war-torn Tigray, Ethiopia. *PLoS ONE* **19**, e0310128 (2024). 39576201 10.1371/journal.pone.0310128 PMC11581278

[42] Ditekemena, J. D. *et al.* COVID-19 vaccine acceptance in the Democratic Republic of Congo: a cross-sectional survey. *Vaccines (Basel)* **9**, 153 (2021). 10.3390/vaccines9020153. 10.3390/vaccines9020153 PMC7917589 33672938

[43] Katonge, J. H. Exploring the role of traditional remedies, cultural practices, and belief interventions in combating COVID-19 in Dodoma City, Tanzania. *Pharmacol. Res. Nat. Prod.* **7**, 100225 (2025).

[44] Ting, R. S., Aw Yong, Y. Y., Tan, M. M. & Yap, C. K. Cultural responses to COVID-19 pandemic: religions, illness perception, and perceived stress. *Front. Psychol.* **12**, 634863 (2021). 34421700 10.3389/fpsyg.2021.634863 PMC8375556

[45] Vederhus, S. et al. Cultural factors influencing COVID-19-related perceptions and behavior, seen from immigrants’ own perspective—a qualitative study in Norway. *Arch. Public Health* **82**, 110 (2024). 39026302 10.1186/s13690-024-01327-z PMC11264612

[46] Sisay, A. L. et al. Barriers and intention to get vaccinated for COVID-19 and associated factors among adults in Southwest Ethiopia: a theory of planned behavior approach. *Infect. Drug Resist.* **16**, 5741–5754 (2023). 37670980 10.2147/IDR.S419952 PMC10476652

[47] Katonge, J. H. Exploring the role of traditional remedies, cultural practices, and belief interventions in combating COVID-19 in Dodoma City, Tanzania. *Pharmacol. Res. Nat. Prod.* **7**, 100225. 10.1016/j.prenap.2025.100225 (2025).

[48] Muluneh, M. D. et al. COVID-19 knowledge, attitudes, and vaccine hesitancy in Ethiopia: a community-based cross-sectional study. *Vaccines* **11**, 774 (2023). 37112686 10.3390/vaccines11040774 PMC10140841

[49] Yoda, T. & Katsuyama, H. Willingness to receive COVID-19 vaccination in Japan. *Vaccines* **9**, 48 (2021). 33466675 10.3390/vaccines9010048 PMC7828811

[50] Shiferie, F., Sada, O., Fenta, T., Kaba, M. & Fentie, A. M. Exploring reasons for COVID-19 vaccine hesitancy among healthcare providers in Ethiopia. *Pan Afr. Med. J.* **40**, 1 (2021). 10.11604/pamj.2021.40.213.30699 PMC8783311 35136476

[51] Benayad, F. Z. et al. Prevalence and predictive determinants of adherence to vaccination against COVID-19 among mothers who gave birth in the last two years in Morocco. *Clin. Epidemiol. Glob. Health* **20**, 101241 (2023). 36743948 10.1016/j.cegh.2023.101241 PMC9884142

[52] Mohamed, A. E. et al. Exploring challenges to COVID-19 vaccination in the Darfur region of Sudan. *Am. J. Trop. Med. Hyg.* **106**, 17–23 (2022). 10.4269/ajtmh.21-0782 PMC8733522 34758448

[53] Mills, M. C. & Salisbury, D. The challenges of distributing COVID-19 vaccinations. *EClinicalMedicine* **31**, 100674 (2021). 33319186 10.1016/j.eclinm.2020.100674 PMC7725651

[54] Forman, R., Shah, S., Jeurissen, P., Jit, M. & Mossialos, E. COVID-19 vaccine challenges: what have we learned so far and what remains to be done?. *Health Policy* **125**, 553–567 (2021). 33820678 10.1016/j.healthpol.2021.03.013 PMC7997052

