# Supplementary material for: A qualitative exploration of deterrents to COVID-19 vaccination uptake among adults in post-war Tigray, Northern Ethiopia
Source: Sci Rep. 2025 Dec 12;16:137. doi: 10.1038/s41598-025-28879-6 (PMC12764872; doi:10.1038/s41598-025-28879-6)
Supplement: Supplementary file 1 — Supplementary Information. [file 41598_2025_28879_MOESM1_ESM.docx]

**A Qualitative Exploration of Deterrents to COVID-19 Vaccination Uptake among Adults in Post-War Tigray, Northern Ethiopia**

Ferehiwot Hailemariam Tesfa^*1^, Znabu Hadush Kahsay^1^, Tadele Tesfean^2^, Adhena Ayaliew^1^, Brhane Ayele^3^, Tsegay Hadgu^3^, Hailay Gebretnsae^3^, Moges Mekonnen^5^, Ataklti Fisseha^5^, Yaynshet Gebreyohannes^5^, Joy Kenyi^5^, Hnin Su Mon^5^, Hayelom Kahsay^3^, Ashenafi Asmelash^6^, Gebrehaweria Gebrekurstos^4^, Mussie Alemayehu^1^, Araya Abrha Medhanyie^1^

***Corresponding Author**: Ferehiwot Hailemariam Tesfa

Email: ferehaile72@gmail.com

**Author information**

Znabu Hadush Kahsay: hadiszinabu@gmail.com

Tadele Tesfean: tesfeantadele@gmail.com

Adhena Ayaliew: adhenaayalew@gmail.com

Brhane Ayele: brhane3127@gmail.com

Tsegay Hadgu: tsegayhadgu28@gmail.com

Hailay Gebretnsae: hailish14@gmail.com

Moges Mekonnen: momekonnen@unicef.org

Ataklti Fisseha: afisseha@unicef.org

Yaynshet Gebreyohannes: ygebreyohannes@unicef.org

Joy Kenyi: jokenyi@unicef.org

Hnin Su Mon: hsmon@unicef.org

Hayelom Kahsay: hayelomk21@gmail.com

Ashenafi Asmelash: ashenafiasmelash@gmail.com

Gebrehaweria Gebrekurstos: gedeftera@gmail.com

Mussie Alemayehu: mossalex75@gmail.com

Araya Abrha Medhanyie: araya.medhanyie@gmail.com

**Annex: Interviewer-Administered Questionnaire**

***Introduction and Consent:***

Hello. My name is _______________________________________. I am working with the joint team from Tigray Regional Health Bureau (TRHB), Mekelle University College of Health Sciences (MUCHS), Tigray Health Research Institute (THRI) and Mums for Mums (MfM). We are now conducting a qualitative on **Behavioral *and social drivers (BeSD)of COVID-19 vaccination, Obstetric fistula (OF) and Pelvic Organ Prolapse (POP), Integrated Measle Campaign (IMC) and Routine Immunization***. The information we collect will help the regional government to plan on the COVID-19 vaccine uptake and intention, assessing the burden of POP and OF, IMC and routine immunization coverage and factors affecting and to take appropriate measures. You are as [**Women or Men]** are selected for the survey. Your participation will take few minutes that could range from 45 minutes to an hour and half. Your participation is completely voluntary and anonymous. All of the answers you give will be confidential and will not be shared with anyone other than members of our survey team. We hope you will agree to answer the questions since your answers to the question are important. If I ask any question you don't want to answer, just let me know and I will go on to the next question or you can stop the interview at any time. In addition, will use a voice recorder to effectively capture your response, thus I can transcribe it latter.

Would you be willing to take the survey?

- Yes
- No

IF THE RESPONSE IS “**YES**” TO THE CONSENT SHEET: Thank you very much. Do you have any questions for me before we begin?

PROCEED TO THE SURVEY SCREENER AFTER ADDRESSING ANY QUESTIONS.

IF THE RESPONSE IS “**NO**” TO THE CONSENT SHEET: Thank you very much. END INTERVIEW

For any questions related to the study, please call the investigators’ phone address Dr. Mussie Alemayehu (0914749082, Mekelle university, college of Health Sciences, Mekelle university.

**COVID Vaccination tool I: FGD guide for adult male and females**

*[Separate FGDs should be conducted for male and female]*

**Interview details and Socio-Demographic information**

Zone: ___________________

Woreda: ________________

Health Facility: ______________

FGD Session Code : _______

Tape Code : _______

Date of Interview:

Interview Start Time: ____________

End time: _____________

Name of Interviewer:

| Participant’s code | Age | Educational status | Marital status | Occupation | For how long have you lived in this place? (in years) |
| --- | --- | --- | --- | --- | --- |
| P1 |  |  |  |  |  |
| P2 |  |  |  |  |  |
| P3 |  |  |  |  |  |
| P4 |  |  |  |  |  |
| P5 |  |  |  |  |  |
| P6 |  |  |  |  |  |
| P7 |  |  |  |  |  |
| P8 |  |  |  |  |  |
| P9 |  |  |  |  |  |
| P10 |  |  |  |  |  |

**COVID Vaccination tool I: FGD guide**

| **Topic focus** | **Core questions** | Probes |
| --- | --- | --- |
| 1. **Thoughts and Feelings** | | |
| **General** | Could you tell something you feel about COVID-19? |  |
| Perceived COVID risk – self | Tell me, how concerned are you about getting COVID?  (Ask personalized risk related to their day-to day activity) | - Why do you feel that way? - How likely do you think it is? - How severe do you think it would be? |
| COVID Vaccine information | Would you tell me what have you heard about the COVID vaccine? | - From whom do you hear about the vaccine? |
| COVID vaccine confidence | How do you think you’ll feel about the COVID vaccine when it becomes available?  Probes: | Could you tell me the benefits you assume for being vaccinated?   - (Relate back to perceived COVID risk, and how important it will be) - Importance in protecting others - Alignment with spiritual or religious beliefs - What about possible side-effects you think? - What are your thoughts/worries about the safety of the vaccine?   - Newness   - Thoughts on whether it will work |

1. **Motivation**

| **Topic focus** | **Core questions** |  |
| --- | --- | --- |
| COVID Vaccine Intention | - Have you thought about getting the COVID vaccine when it becomes available? Why/ why not? | - Could you tell me the reason that motivates you to use the vaccine? - What about reasons to not vaccinated? |

1. **Social Processes**

| **Topic focus** | **Core questions** | Probe |
| --- | --- | --- |
| COVID Vaccine – decision process | Would you tell me how you would reach in the decision to get vaccinated for COVID-19? | - Would there be anyone else involved in the decision? - Who do you think you might discuss it with? |
| COVID vaccine- Descriptive and norms | If a COVID vaccine is recommended by health care workers, would you tell me what other people around you will do/think? | - Family and friends - Religious or community leaders recommend? - How will their thought affect your intention? |
| Provider recommendation | - Could you tell me what care providers recommend about COVID-19 vaccine? - How do you personally see the provider’s recommendation on COVID-19 vaccine? |  |

1. **Practical Issues**

| **Topic focus** | **Core questions** |  |
| --- | --- | --- |
| Ever gone to get vaccines | Have you ever had a vaccine as an adult? | What did you think was good about the vaccine? What about bad about it? |
| COVID vaccine-availability, access affordability, service satisfaction  And service quality | Say, for example, you were to decide to get a COVID vaccine; can you tell me, how you would get (got) vaccinated? | Start at the beginning  Probe:   - Would you need to ask permission? - Where would you go to get it? - How would you get there? - What other things would you need to do (e.g. find care for young children, find someone to take care of livelihood / get up earlier to take care of household duties) - Would there be any cost involved for you (not just for vaccine, but things like transport) - How much do you trust the health care worker who will give you the vaccine? - What would make it easy for you to get a COVID vaccine if it was recommended and available? |

**Is there anything else you’d like to say?**

***The End!***

**COVID Vaccination tool II: IDI guide**

**Interview details and Socio-Demographic information**

Zone: ___________________

Woreda: ________________

Health Facility: ______________

FGD Session Code : _______

Tape Code : _______

Date of Interview:

Interview Start Time: ____________

End time: _____________

Name of Interviewer: __________

| Participant’s code | Age | Educational status | occupation | Number of years lived in the area |
| --- | --- | --- | --- | --- |
| P1 |  |  |  |  |

| **Topic focus** | **Core questions** | Probes |
| --- | --- | --- |
| **1)Thoughts and Feelings** | | |
| **General** | Could you tell something you feel about COVID-19? |  |
| Perceived COVID risk – self | Tell me, how concerned are you about getting COVID?  (Ask personalized risk related to their day-to day activity) | - Why do you feel that way? - How likely do you think it is? - How severe do you think it would be? |
| COVID Vaccine information | Would you tell me what have you heard about the COVID vaccine? | - From whom do you hear about the vaccine? |
| COVID vaccine confidence | How do you think you’ll feel about the COVID vaccine when it becomes available?  Probes: | Could you tell me the benefits you assume for being vaccinated?   - (Relate back to perceived COVID risk, and how important it will be) - Importance in protecting others - Alignment with spiritual or religious beliefs - What about possible side-effects you think? - What are your thoughts/worries about the safety of the vaccine?   - Newness   - Thoughts on whether it will work |

1. **Motivation**

| **Topic focus** | **Core questions** |  |
| --- | --- | --- |
| COVID Vaccine Intention | - Have you thought about getting the COVID vaccine when it becomes available? Why/ why not? | - Could you tell me the reason that motivates you to use the vaccine? - What about reasons to not vaccinated? |

1. **Social Processes**

| **Topic focus** | **Core questions** | Probe |
| --- | --- | --- |
| COVID Vaccine – decision process | Would you tell me how you would reach in the decision to get vaccinated for COVID-19? | - Would there be anyone else involved in the decision? - Who do you think you might discuss it with? |
| COVID vaccine- Descriptive and norms | If a COVID vaccine is recommended by health care workers, would you tell me what other people around you will do/think? | - Family and friends - Religious or community leaders recommend? - How will their thought affect your intention? |
| Provider recommendation | - Could you tell me what care providers recommend about COVID-19 vaccine? - How do you personally see the provider’s recommendation on COVID-19 vaccine? |  |

1. **Practical Issues**

| **Topic focus** | **Core questions** |  |
| --- | --- | --- |
| Ever gone to get vaccines | Have you ever had a vaccine as an adult? | What did you think was good about the vaccine? What about bad about it? |
| COVID vaccine-availability, access affordability, service satisfaction  And service quality | Say, for example, you were to decide to get a COVID vaccine; can you tell me, how you would get (got) vaccinated? | Start at the beginning  Probe:   - Would you need to ask permission? - Where would you go to get it? - How would you get there? - What other things would you need to do (e.g. find care for young children, find someone to take care of livelihood / get up earlier to take care of household duties) - Would there be any cost involved for you (not just for vaccine, but things like transport) - How much do you trust the health care worker who will give you the vaccine? - What would make it easy for you to get a COVID vaccine if it was recommended and available? |

**Is there anything else you’d like to say?**

***The End!***

**COVID Vaccination tool III: KII Guide for HWs**

**Interview details and Socio-Demographic information**

Zone: ___________________

Woreda: ________________

Health Facility: ______________

FGD Session Code : _______

Tape Code : _______

Date of Interview:

Interview Start Time: ____________

End time: _____________

Name of Interviewer:

| Participant’s code | Age | Educational status | Types of Profession | Total work experience (in years) | Current position in the HF |
| --- | --- | --- | --- | --- | --- |
| P1 |  |  |  |  |  |

1. **Thoughts and Feelings**

| **Topic focus** | **Core questions** | Probes |
| --- | --- | --- |
| **General** | Could you tell something you about COVID-19? | |
| Perceived COVID risk – self | Tell me, how concerned are you about getting COVID?  (Ask personalized risk related to their day-to day activity) | - Why do you feel that way? - How likely do you think it is? - How severe do you think it would be? |
|  | Tell me what you think about the risk that you could give COVID to your patients? |  |
| COVID stigma | Being a health care worker, how are you usually treated by others in the community in reference to COVID-19? | - Have you noticed any form of discrimination since the pandemic? |
| COVID Vaccine information | Would you tell me what have you heard about the COVID vaccine? | - From whom do you hear about the vaccine? |
| COVID vaccine confidence | How do you think you’ll feel about the COVID vaccine when it becomes available?  Probes: | Could you tell me the benefits you assume for being vaccinated?   - (Relate back to perceived COVID risk, and how important it will be) - Importance in protecting others - Alignment with spiritual or religious beliefs - What about possible side-effects you think? - What are your thoughts/worries about the safety of the vaccine?   - Newness   - Thoughts on whether it will work |

1. **Motivation**

| **Topic focus** | **Core questions** |  |
| --- | --- | --- |
| COVID Vaccine Intention | - Have you thought about getting the COVID vaccine when it becomes available? Why/ why not? | - Could you tell me the reason that motivates you to use the vaccine? - What about reasons to not vaccinated? |

1. **Social Processes**

| **Topic focus** | **Core questions** | Probe |
| --- | --- | --- |
| COVID Vaccine – decision process | Would you tell me how you would reach in the decision to get vaccinated for COVID-19? | - Would there be anyone else involved in the decision? - Who do you think you might discuss it with? |
| COVID vaccine – safe to see family and friends | Would you tell me how being vaccinated could change/ your daily-to day activities? | - Going out in public - Attending social/religious events - Going back to work/school |
| COVID vaccine- Descriptive and norms | If a COVID vaccine is recommended by health care workers, would you tell me what other people around you will do/think? | - Family and friends - Religious or community leaders recommend? - How will their thought affect your intention? |
| Provider recommendation | - As a care provider, what do you recommend about COVID-19 vaccine? - How do you evaluate the acceptance of community for the provider’s recommendation on COVID-19 vaccine? |  |

1. **Practical Issues**

| **Topic focus** | **Core questions** |  |
| --- | --- | --- |
| Ever gone to get vaccines | Have you ever had a vaccine as an adult? | What did you think was good about the vaccine? What about bad about it? |
| COVID vaccine-availability, access affordability, service satisfaction  And service quality | Say, for example, you were to decide to get a COVID vaccine; can you tell me, how you would get (got) vaccinated? | Start at the beginning  Probe:   - Would you need to ask permission? - Where would you go to get it? - How would you get there? - What other things would you need to do (e.g. find care for young children, find someone to take care of livelihood / get up earlier to take care of household duties) - Would there be any cost involved for you (not just for vaccine, but things like transport) - How much do you trust the health care worker who will give you the vaccine? - What would make it easy for you to get a COVID vaccine if it was recommended and available? |

**Is there anything else you’d like to say?**

**The End!**
